# Characterization and Comparison of WO_3_/WO_3_-MoO_3_ and TiO_2_/TiO_2_-ZnO Nanostructures for Photoelectrocatalytic Degradation of the Pesticide Imazalil

**DOI:** 10.3390/nano13182584

**Published:** 2023-09-18

**Authors:** Mireia Cifre-Herrando, Gemma Roselló-Márquez, Pedro José Navarro-Gázquez, María José Muñoz-Portero, Encarnación Blasco-Tamarit, José García-Antón

**Affiliations:** Ingeniería Electroquímica y Corrosión (IEC), Instituto Universitario de Seguridad Industrial, Radiofísica y Medioambiental (ISIRYM), Universitat Politècnica de València, C/Camino de Vera s/n, 46022 Valencia, Spain; mcifher@upvnet.upv.es (M.C.-H.); gemromar@etsii.upv.es (G.R.-M.); pednagz@etsii.upv.es (P.J.N.-G.); mjmunoz@iqn.upv.es (M.J.M.-P.); meblasco@iqn.upv.es (E.B.-T.)

**Keywords:** nanostructures, hybrid nanostructures, WO_3_, TiO_2_, water splitting, emerging contaminants

## Abstract

Tungsten oxide (WO_3_) and zinc oxide (ZnO) are n-type semiconductors with numerous applications in photocatalysis. The objective of this study was to synthesize and characterize different types of nanostructures (WO_3_, WO_3_-Mo, TiO_2_, and TiO_2_-ZnO) for a comparison of hybrid and pure nanostructures to use them as a photoanodes for photoelectrocatalytic degradation of emerging contaminants. With the aim of comparing the properties of both samples, field emission scanning electron microscopy (FE-SEM) and confocal laser-Raman spectroscopy were used to study the morphology, composition, and crystallinity, respectively. Electrochemical impedances, Mott-Schottky, and water splitting measurements were performed to compare the photoelectrochemical properties of photoanodes. Finally, the photoelectrocatalytic degradation of the pesticide Imazalil was carried out with the best optimized nanostructure (TiO_2_-ZnO).

## 1. Introduction

Water pollution is one of today’s major global concerns, with drinking water quality being one of the priorities of the World Health Organization [1]. This problem is compounded by the increase in organic compounds derived from human activities. Many of these compounds are persistent organic pollutants, such as pesticides or pharmaceuticals, that cannot be removed from water with conventional processes [2,3]. In order to eliminate these types of pollutants, advanced oxidation processes are being studied [4,5,6]. Among them, photoelectrocatalysis has been demonstrated as a promising method for water remediation [7,8,9].

Hence, photoelectrocatalysis (PEC) is an advanced oxidation process that combines electrolytic and photocalytic processes in which there is a control of the system potential, which allows improving its overall performance. Figure S1 (see Supplementary Material) shows a graphic scheme of the process. In the PEC process, electron-hole pairs ($e_{CB}^{-}/h_{VB}^{+})$ are generated by UV-Vis irradiation of the semiconductor and the application of an external low potential. The process continues with the formation of extremely reactive radicals, hydroxyl radicals (·OH), on the semiconductor surface. Lastly, hydroxyl radicals (·OH) are used as an oxidizing agent to degrade organic pollutants, such as EDCs [7,10].

For an efficient PEC process, it is necessary to find a suitable semiconductor photoelectrode. Oxide semiconductors are the most common materials, in particular those with high visible-light absorption, efficient charge carrier separation, and chemical stability, which could be TiO_2_ or WO_3_ [11].

On one hand, WO_3_ is claimed to be a proper material for PEC applications due to its high resistance to photocorrosion, stability in acidic media, good electron transport properties, and band gap (Eg = 2.6 eV, λ = 480 nm). As its band gaps are capable of only capturing 12% of the incident light of the solar spectrum, several approaches have been tried to enhance the PEC of WO_3_ by band-gap modifications [12]. Doping of WO_3_ with Mo can narrow the band gap of WO_3_ and, consequently, improve its photocatalytic properties [13]. Therefore, a simple method for the synthesis of hybrid WO_3_-MoO_3_ nanostructures is proposed.

On the other hand, TiO_2_ is one of the most extensively studied materials for PEC [14]. This is because it is a non-toxic semiconductor, has high chemical stability, excellent photocatalytic activity, significant cost-effectiveness, and a capacity to generate electron/hole pairs [15]. However, its photocatalytic applications are limited to ultraviolet irradiation due to its wide band gap (3.2 eV, λ = 390 nm) [16]. In order to reduce its bandgap, different elements could be added to TiO_2_ nanostructures. In this study, hybrid nanostructures of TiO_2_ with ZnO are synthesized to increase the TiO_2_ photoelectrochemical properties.

Therefore, the enhanced nanostructures obtained are applied as photoanodes for PEC degradation of contaminants in water. The contaminant chosen to evaluate the effectiveness of nanostructures in PEC degradation is Imazalil. Imazalil is a pesticide widely used in the fruit and vegetable industry to combat fungal diseases preventively or during their initial stages. Its range of application is wider than that of most fungicides since it is effective against strains of fungi and molds that are resistant to other pesticides [17]. However, its massive use has favored the appearance of highly resistant strains, which are used in significantly higher doses and accumulate in the peel of the fruit. Regarding its properties, it is a moderately soluble compound (1400 mg/L), thermally stable up to approximately 285 °C, and chemically stable at room temperature, with a useful life of more than 2 years in the absence of light. Regarding its toxicity, Imazalil is classified as “probably carcinogenic to humans”. It is characterized as a category II (moderately dangerous product) for both oral and dermal toxicity [18]. Therefore, it is considered a potentially dangerous pollutant, and it would be interesting to degrade it using efficient methods such as PEC.

Thus, the objective of this work is to synthesize and characterize different types of nanostructures (WO_3_, hybrid WO_3_-MoO_3_, TiO_2_, and hybrid TiO_2_-ZnO) for a comparison of hybrid and pure nanostructures. Then, the optimized nanostructure will be used as photoanodes for PEC degradation to remove contaminants from wastewater specifically, the pesticide Imazalil.

## 2. Materials and Methods

### 2.1. Synthesis of Nanostructures

The procedure to synthesize nanostructures was conducted by electrochemical anodization under hydrodynamic conditions using a rotatory disk electrode (RDE). The process was optimized by the authors in previous works [19,20].

For the WO_3_ nanostructures, anodization of W was carried out at a velocity of 375 rpm, applying 20 V for 4 h. The electrolyte consisted of 1.5 M methanosulfonic acid and 0.01 M citric acid at 50 °C. After anodization, WO_3_ nanostructures were annealed for 4 h at 600 °C in an air atmosphere.

For synthesizing hybrid nanostructures of WO_3_-MoO_3_, the same anodization was carried out, but different concentrations of Na_2_MoO_4_·2H_2_O (Mob) were added to the electrolyte.

For the TiO_2_, the electrochemical anodization of Ti was carried out at room temperature under 3000 rpm, applying 30 V for 3 h. The electrolyte consisted of glycerol (60% vol.), water (40% vol.), and 0.27 M NH_4_F. Finally, the samples were heated at a temperature of 450 °C for 1 h in an air atmosphere to transform TiO_2_ nanostructures into the anatase phase.

For the TiO_2_-ZnO hybrid nanostructures, after forming TiO_2_ nanosponges, the ZnO electrodeposition technique was performed from a Zn(NO_3_)_2_ solution at 75 °C using a potential of −0.86 V_Ag/AgCl_ for 15 min in an Autolab PGSTAT302N potentiostat. A quartz reactor with a three-electrode configuration was used: the working electrode was the nanostructure synthesized, the reference electrode was an Ag/AgCl (3 M KCl) electrode and the counter electrode was a platinum wire. The effect of Zn(NO_3_)_2_ concentration (1–10 mM) on the photoelectrochemical properties of the photoelectrode was analyzed.

### 2.2. Morphological and Crystalline Characterization

Field emission scanning electron microscopy (FE-SEM) with energy-dispersive X-ray spectroscopy (EDX) permitted the study of the morphology and the identification of the elements present in the synthesized nanostructures. The equipment used was a Zeiss Ultra-55 scanning electron microscope (Jena, Germany) at 20 kV. Furthermore, the crystallinity of the nanostructure was analyzed by a Raman confocal laser spectroscopy (Witec alpha 300R, Ulm, Germany) with a neon laser of 488 nm at 420 μW and also by X-ray diffraction (XRD) using a D8AVANCE diffractometer (Bruker, MA, USA) equipped with a monochromatic Cu Kα1 source.

### 2.3. Photoelectrochemical Properties

A potentiostat (Autolab PGSTAT302N, Metrohm, Herisau, Switzerland) and a solar simulator (500 W xenon lamp) were used to study the photoelectrochemical properties of the samples. The reactor and configuration used were the same as for the ZnO deposition. Photoelectrochemical impedance spectroscopy (PEIS) tests were performed to analyze the electrochemical and photoelectrochemical behavior of each synthesized sample. For the WO_3_ samples, the tests were carried out applying simulated sunlight with the simulator mentioned before and a potential of 1 V_Ag/AgCl_ in a frequency range of 100 kHz to 10 mHz with a signal amplitude of 10 mV. The electrolyte used was 0.1 M H_2_SO_4_. On the other hand, for the TiO_2_ nanostructures, the applied potential was 0.6 V_Ag/AgCl_ and the electrolyte was 0.1 M NaOH, the rest of the conditions being the same. For water splitting tests, a potential sweep with a scan speed of 2 mV·s^−1^ was carried out, applying dark (30 s) and light (10 s) cycles.

### 2.4. Photoelectroatalytic Degradation

The PEC degradation was carried out starting from a 10 ppm solution of Imazalil under lighting conditions (AM 1.5) at a potential of 0.6 V_Ag/AgCl_ for 24 h. The electrodes used were TiO_2_/ZnO hybrid nanostructures as photoanodes, an Ag/AgCl (3 M KCl) reference electrode, and a platinum wire as a counter electrode. The electrolyte of the Imazalil was optimized. The electrolyte of the first degradation was 0.1 M NaOH (pH = 13), and the optimized electrolyte was 0.1 M Na_2_SO_4_ (pH = 6.2).

The degradation course was monitored by ultra high performance liquid chromatography and mass spectrometry (UHPLC-MS-QTOF). The equipment used was an Agilent 1290 Infinity equipped with a C-18 analytical column (Agilent ZORBAX Eclipse Plus, Santa Clara, CA, USA) of 50 mm × 2.1 mm with a particle size of 1.8 μm. The mobile phases were water (A) and acetonitrile (B), both acidified with 0.1% (*v*/*v*) acetic acid. The experimental conditions of the equipment were the following: injection volume of 0.2 µL, flow rate of 1 mL·min^−1^, and column temperature of 45 °C. Furthermore, the conditions for the mass spectrometer were: positive ionization, capillary voltage 4000 V, nebulizer pressure 40 psi, gas temperature 325 °C, skimmer voltage 65 V, octopolar rf 250 V, and voltage fragmentor 190 V.

## 3. Results and Discussion

### 3.1. Morphologycal and Crystallyne Characterization

#### 3.1.1. FE-SEM

**Figure 1:**
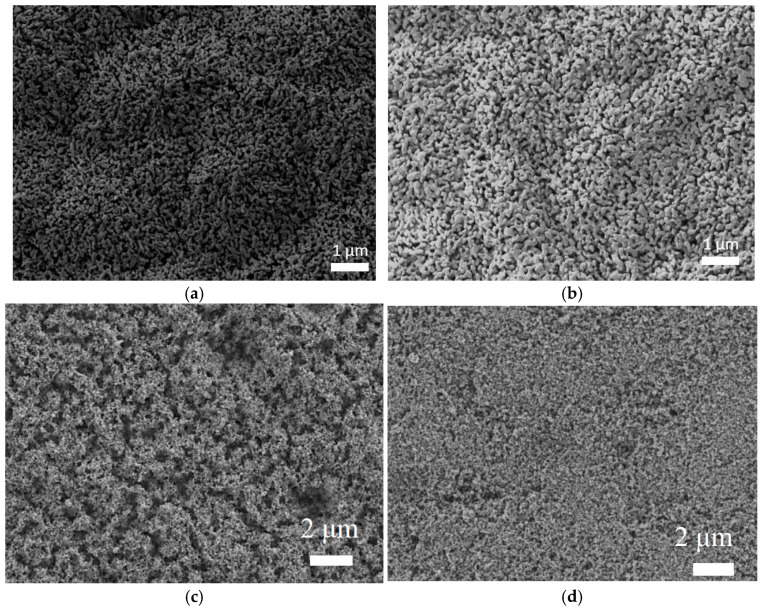
FE-SEM images of the different nanostructures (**a**) WO_3_ (**b**) WO_3_ + 0.01 M Mob (**c**) TiO_2_ and (**d**) TiO_2_ + 0.01 M Zn(NO_3_)_2_.

Figure 1 presents the FE-SEM images of the synthesized nanostructures. Observing the images in Figure 1a,b, the effect of doping the WO_3_ nanostructures with MoO_3_ can be observed. In both cases, defined, small-sized nanoparticles with a mountain shape were obtained. In contrast, Figure 1c,d allows us to compare the effect of adding ZnO to the TiO_2_ nanostructures. Figure 1c shows nanostructures with a rough surface and high specific area, typical for a nanosponge-like morphology [21]. Figure 1d shows the overall appearance of the hybrid nanostructures TiO_2_-ZnO, where a nanosponge-shaped nanostructure without the existence of different particles on its surface can be observed. Consequently, the morphology is the same for the pure nanostructure as for the hybrid. This shows that no MoO_3_ or ZnO agglomerations happened during the doping procedure [15].

#### 3.1.2. EDX

EDX analysis has been carried out to demonstrate the occurrence of MoO_3_ and ZnO in the nanostructures and to quantify the elements. Table 1 shows the results of the EDX analysis for both hybrid nanostructures. In the case of MoO_3_ addition, the percentage of MoO_3_ increases when increasing the concentration in the electrolyte. Similarly, for the ZnO deposition, the quantity of Zn in the samples increases by increasing the concentration of Zn(NO_3_)_2_ in the electrodeposition. Consequently, it can be affirmed that MoO_3_ and ZnO are deposited over the pure nanostructure.

#### 3.1.3. Raman

**Figure 2:**
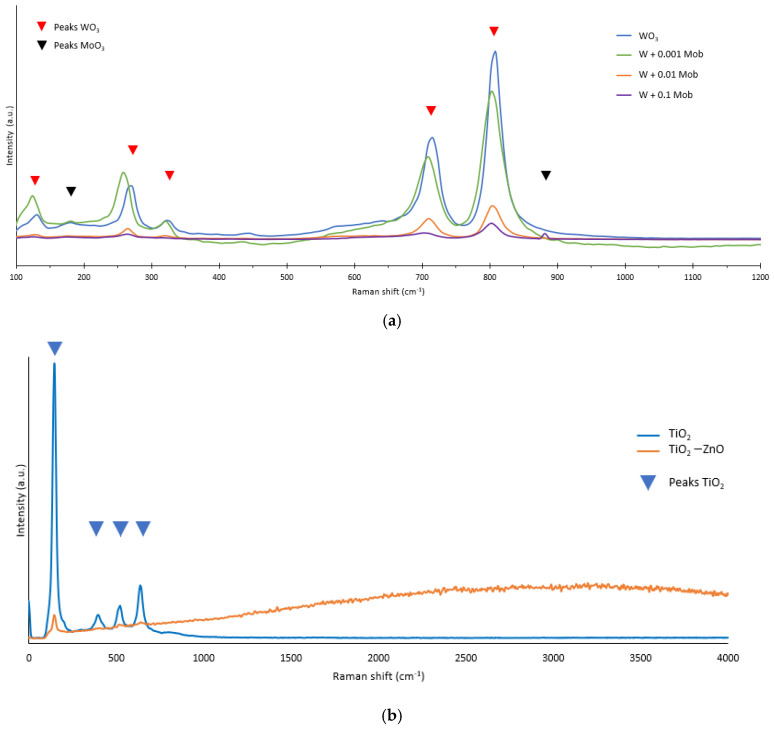
Raman spectra of the nanostructures (**a**) WO_3_ + different Mob concentrations and (**b**) TiO_2_ and TiO_2_-ZnO synthesized with 0.01 M Zn(NO_3_)_2_.

The Raman spectra of the different nanostructures are presented in Figure 2. Figure 2a shows the Raman spectra of the WO_3_ nanostructure and the hybrid WO_3_-MoO_3_ nanostructures after a heat treatment of 600 °C for 4 h. The shape of the spectra for all tungsten samples is similar. The blue line represents the pure WO_3_ nanostructure, where it can be clearly seen the peaks corresponding to the monoclinic WO_3_ phase: 135, 270, 327, 714, and 805 cm^−1^ [22,23,24,25]. The sharpest peak is the one that appears at 804 cm^−1^, which corresponds to ν(O-W^6+^-O) stretching mode. After this, the peak is at 714 cm^−1^, which is assigned to the ν(O-W-O) mode. The other peaks (327 cm^−1^, 270 cm^−1^, and 135 cm^−1^) are assigned respectively to the vibration of the O-W^5+^-O, the bending vibration δ(O-W-O). and to lattice modes [25,26,27]. Furthermore, in Figure 2a, the spectra of the hybrid nanostructures WO_3_-MoO_3_ (lines green, orange, and purple) is shown. It can be observed that when Mob is added to the electrolyte, some of the principal bands of MoO_3_ can be appreciated: 190 and 867 cm^−1^ [28]. The peak at 867 cm^−1^ corresponds to the stretching vibration of MoO_3_, and the weak bands below 200 cm^−1^ are assigned to mixed lattice modes of MoO_3_ and WO_3_ [25]. From Figure 2a, it can also be appreciated that the relative intensity of the bands corresponding to the monoclinic WO_3_ phase diminishes with increasing percentage of Mob in the electrolyte, along with a slight shift in the peak positions. This is because when molybdate is added to the electrolyte, the nanostructure is not only composed of WO_3_, but MoO_3_ is also deposited on the structure, as verified by FESEM images and in EDX analysis, and the peaks corresponding to WO_3_ were weaker due to a minor percentage of WO_3_ composition in the hybrid nanostructure [28].

Regarding TiO_2_ samples, Figure 2b shows the spectra of the TiO_2_ nanostructures after a heat treatment of 450 °C for 1 h. For the pure nanostructure (blue line), the peaks associated with the anatase phase of TiO_2_ are observed (145, 397, 520, and 635 cm^−1^) [29,30,31,32]. In Figure 2b, it can be appreciated that the high peak is that one at 145 cm^−1^. This peak is the most characteristic of the anatase phase of TiO_2_ and arises from the external vibration of its structure. However, for the hybrid nanostructure with ZnO, the peaks are not observed due to the high fluorescence of ZnO. Therefore, this allows us to reaffirm that ZnO is indeed deposited on the TiO_2_ nanostructure.

#### 3.1.4. X-ray Diffraction (XRD)

**Figure 3:**
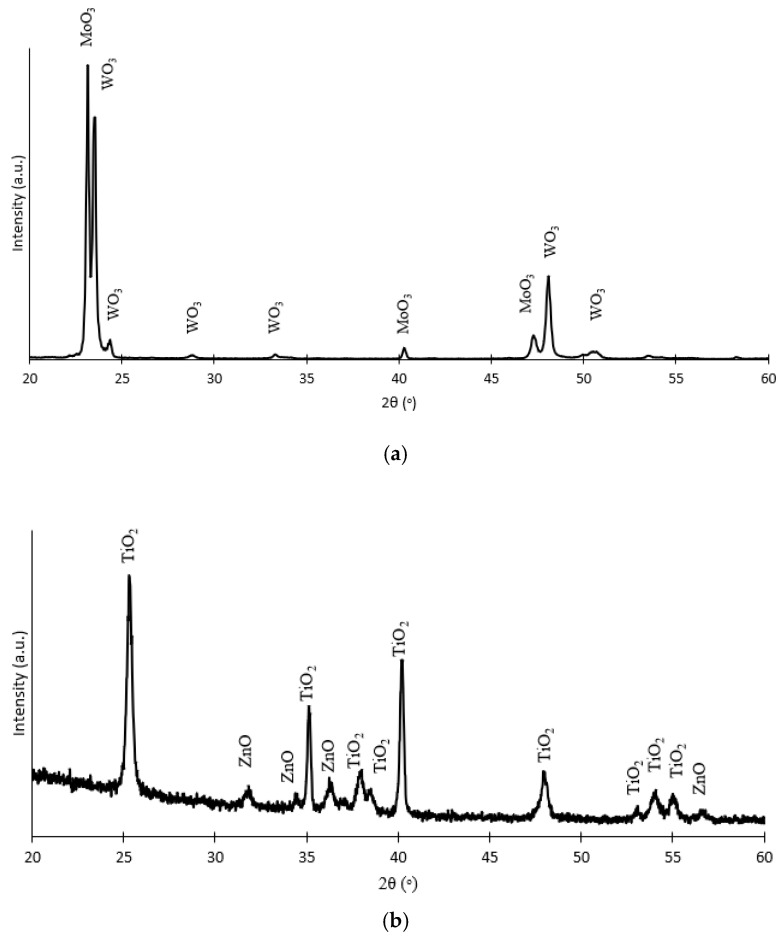
XRD patterns of the hybrid nanostructures (**a**) WO_3_-MoO_3_ synthesized with 0.001 M Mob and (**b**) TiO_2_-ZnO synthesized with 0.01 M Zn(NO_3_)_2_.

X-ray diffraction (XRD) is an analytical technique that allows for the determination of the crystalline phases of the compounds that form the nanostructures. In order to simplify the results obtained, only the XRD pattern of the best hybrid nanostructures is presented. That is the hybrid WO_3_ nanostructure synthesized with 0.001 M Mob (Figure 3a) and the TiO_2_ nanostructure synthesized by depositing 0.01 M Zn(NO_3_)_2_ (Figure 3b).

The XRD patterns for the hybrid nanostructure WO_3_-MoO_3_ are shown in Figure 3a, where peaks both from WO_3_ and MoO_3_ can be appreciated. Diffraction peaks corresponding with the crystalline planes of the monoclinic phase of WO_3_ (0 2 0), (2 0 0), (1 1 2), (0 2 2), (2 2 0), and (2 1 1) are observed at 23.5°, 24.3°, 28.8°, 33.4°, 48.0°, and 50.5° [33,34,35,36]. In addition, peaks from the orthorhombic MoO_3_ phase are also appreciated: 23.1° and 40.0°, corresponding to the planes (1 1 0) and (0 6 0), respectively [25,37,38].

Regarding the nanostructures with Ti, Figure 3b shows the XRD pattern of the hybrid nanostructures TiO_2_-ZnO, where peaks both from TiO_2_ and ZnO can be appreciated. From Figure 3b, it can be appreciated that the most intense peak is that located at 25.3°, which corresponds to the anatase TiO_2_ phase (1 0 1) [39,40]. Furthermore, different peaks of the wurtzite structure of ZnO appear at 31.8°, 34.4°, 36.2°, and 56.7°, corresponding to crystallographic planes (1 0 0), (0 0 2), (1 0 1), and (1 1 0), respectively [40,41].

Furthermore, using the information from the XRD analysis, the crystallite size for the most intense peaks obtained in the XRD profile for each sample has been calculated by using Scherrer’s equation (Equation (1)):

(1) $$D=\frac{k\cdot \lambda }{\mathrm{FWHM}\cdot \cos\theta }$$

where D is the crystallite size, k is a shape factor (0.9), λ is the X-ray wavelength (0.15406 nm), FWHM is the full width at half maximum intensity (expressed in radians), and θ is the Bragg angle at which the crystallite size is calculated (also expressed in radians). Table S1 shows the average crystal size of the pure samples and the hybrid nanostructures. Thus, it can be affirmed that the size of the crystal of the monoclinic phase of WO_3_ (110) is not affected by the deposition of MoO_3_ and likewise, the average crystal size of TiO_2_ was about 26 nm regardless of whether they are TiO_2_ nanosponges or hybrid TiO_2_-ZnO.

Consequently, from the XRD analysis, it is confirmed the correct deposition of the MoO_3_ on the WO_3_ nanostructures and ZnO on the TiO_2_ nanostructures.

### 3.2. Photoelectrochemical Characterization

#### 3.2.1. PEIS Tests

PEIS measurements were carried out under irradiation conditions in order to study the influence of the doping element concentration on the photoelectrochemical properties of the sample, specifically on the separation of the electron/holes generated.

**Figure 4:**
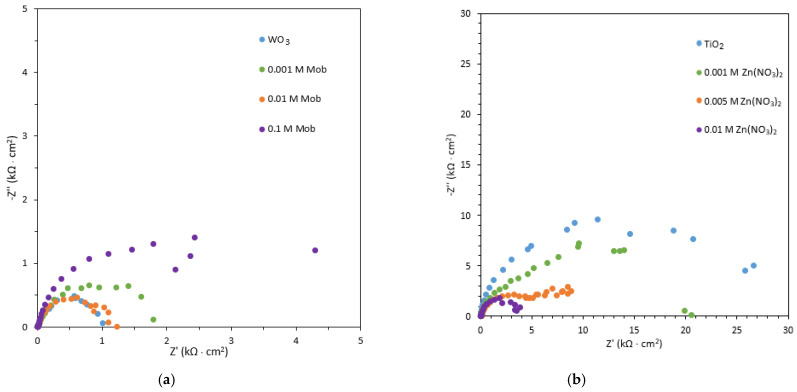
Nyquist plots of the (**a**) WO_3_ nanostructures synthesized with different Mob concentrations and (**b**) TiO_2_ nanostructures synthesized with different Zn(NO_3_)_2_ concentrations.

Nyquist plots are shown in Figure 4a for WO_3_ and Figure 4b for TiO_2_. They are used to study the interfaces between the anode and the electrolyte and to calculate the resistance of holes to charge transfer [42,43]. In all cases, a semicircle can be observed, which is related to the charge transfer resistance of the nanostructure. For WO_3_ nanostructures, as observed in Figure 4a, the Mo-doped WO_3_ nanostructures gave rise to a bigger semicircle than undoped WO_3_ samples. When adding 0.001 M Mob and 0.1 M Mob, the semicircle increases. However, it decreases when adding 0.01 M Mob. This indicated that the WO_3_ and the WO_3_ with 0.01 M Mob nanostructures present low charge transfer resistance between the electrolyte and the electrode interface, leading to better photoelectrochemical properties [44]. For TiO_2_ nanostructures (Figure 4b), as the concentration of the deposited zinc increases, the semicircle decreases, and, therefore, the charge transfer resistance decreases. Being the nanostructures deposited with 0.01 M Zn(NO_3_)_2_, those with lower resistance thus show enhanced electronic transport and charge separation. This improvement is due to the interconnection of the valence and conduction bands of TiO_2_ and ZnO [45].

**Figure 5:**
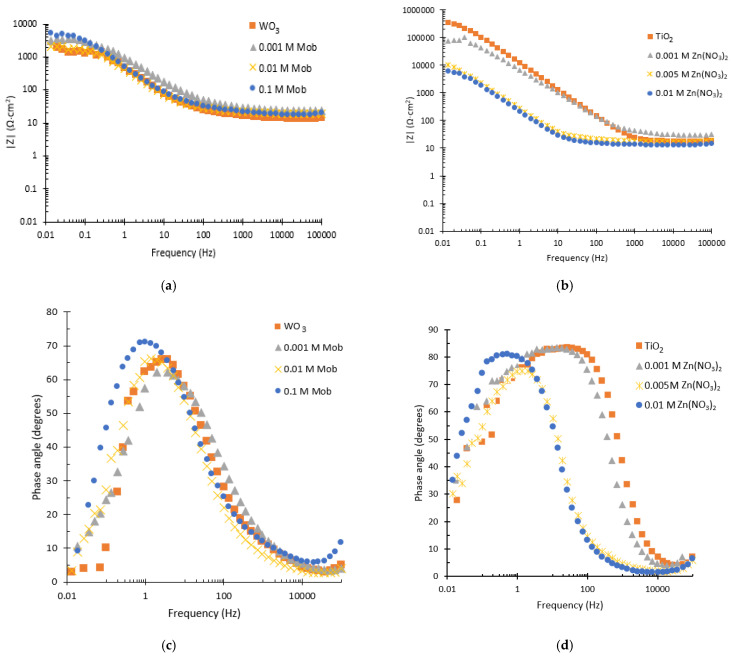
Bode plots of modulus for (**a**) WO_3_ nanostructures synthesized with different Mob concentrations and (**b**) TiO_2_ nanostructures synthesized with different Zn(NO_3_)_2_ concentrations. Bode plots of phase for (**c**) WO_3_ nanostructures synthesized with different Mob concentrations and (**d**) TiO_2_ nanostructures synthesized with different Zn(NO_3_)_2_ concentrations.

Figure 5 shows the plots of Bode modulus and Bode phase for the synthesized nanostructures. From the Bode modulus diagram, for both tungsten and titanium nanostructures (Figure 5a,b), it can be seen that at high frequencies (10^3^–10^5^ Hz), the resistance is similar regardless of the concentration deposited. This is due to the fact that the impedance modulus at high frequencies is related to the resistance of the electrolyte. For low frequencies, however, there is a difference in resistance. In the case of tungsten, it is observed from Figure 5a that all nanostructures are quite similar, although for the pure nanostructure (WO_3_) and the one synthesized with 0.01 M Mob, the resistance decreases, coinciding with the Nyquist results. On the other hand, for the case of titanium, it is observed that as the amount of deposited Zn increases, the resistance decreases, which is also consistent with Nyquist results.

Regarding the Bode phase graphs (Figure 5c,d), in all cases there appears a broad peak that is associated with the superposition of two peaks, which are related to the existence of two time constants (one at high frequencies and the other at intermediate frequencies) [46]. For the tungsten nanostructures, a similar value of phase angle is observed for all the nanostructures except for the one synthesized with 0.1 M Mob, for which a higher value is obtained, indicating higher impedances. For the titanium nanostructures (Figure 5d), a shift to lower frequency values is observed with the deposition of ZnO, which indicates a higher life time of the electron/hole pairs generated [47].

In order to analyze quantitatively the EIS results, the data were fitted using an electrical equivalent circuit (Figure S2, see Supplementary Material). The equivalent circuit is formed by the electrolyte resistance (Rs) coupled with two R-CPE time constants. R_1_-CP_1_ time constant is associated with the recombination of electron/hole pairs, and R_2_-CPE_2_ is associated with the hole transfer from the photoanode to the electrolyte [48,49]. Table 2 lists the simulated impedance parameters calculated from the equivalent circuit. For WO_3_, it can be observed that adding 0.001 M Mob to the electrolyte increases the resistance to charge transfer, adding 0.01 M decreases it again, being inferior to the original nanostructure, and increasing the quantity of Mob up to 0.1 M drastically increases the resistance. These results indicate that the Mo-doped WO_3_ photoanode with 0.01 M and the original WO_3_ photoanode have the lowest charge transfer resistance, which leads to improved electrochemical properties. With higher concentrations of Mob, the resistance increases because the Mo could be deposited on the surface of the nanostructure and prevent electron transfer. For TiO_2_, when forming TiO_2_/ZnO hybrid nanosponges, the charge transfer resistance decreases significantly, increasing the electrical conductivity and electron transfer on the nanostructure. This could be due to the higher number of defects on the surface of the hybrid nanostructure as a consequence of the introduction of ZnO into the nanostructure, obtaining a more efficient charge separation and a greater number of charge carriers.

#### 3.2.2. Mott-Schottky Tests

**Figure 6:**
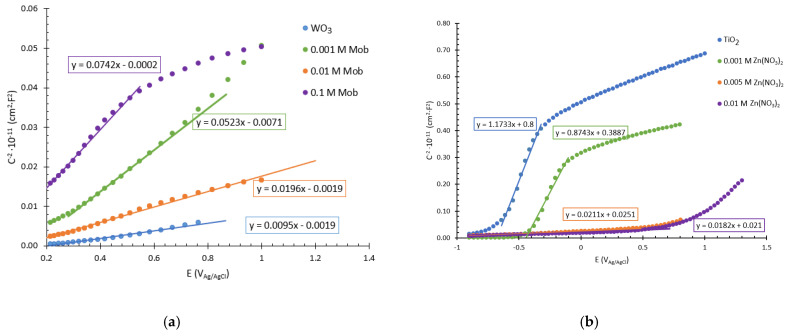
Mott-Schottky plots for (**a**) WO_3_ nanostructures synthesized with different Mob concentrations and (**b**) TiO_2_ nanostructures synthesized with different Zn(NO_3_)_2_ concentrations.

Moreover, Mott-Schottky (MS) tests under irradiation conditions were also performed in order to analyze the electrochemical capacitance in the semiconductor/electrolyte interface of the nanostructure and to calculate the donor density of the different nanostructures. Figure 6 shows the MS plots for (a) WO_3_ nanostructures with molybdenum oxide and (b) TiO_2_ nanostructures with zinc oxide, where there is a positive slope in all cases, characteristic of n-type semiconductors [50]. Table 3 shows the values of the donor density (N_D_) of all samples, calculated from the slope of the MS plots. A high density of charge donors results in high electron and hole mobility within the nanostructures; thus, a greater number of electrochemical reactions can be carried out, so that the photoelectrochemical activity of these photocatalysts will be considerably higher [51,52].

Regarding WO_3_ nanostructures, the N_D_ values obtained are 297.4 × 10^19^ cm^−3^ for the original nanostructure and 54.0 × 10^19^ cm^−3^, 144.1 × 10^19^ cm^−3^, and 37.1 × 10^19^ cm^−3^ for the hybrid nanostructure obtained with 0.001 M, 0.010 M, and 0.1 M Mob, respectively. A higher number of N_D_ is related to a higher number of defects within the nanostructure; consequently, higher electrical conductivities and charge transfer processes are favored. These results are consistent with the previous results obtained from PEIS tests and indicate that the optimum nanostructures are the original nanostructure and the one obtained with 0.010 M Mob. For the Ti nanostructures, the N_D_ values obtained are 1.2 × 10^19^ cm^−3^ for the original nanostructure and 2.1 × 10^19^ cm^−3^, 85.8 × 10^19^ cm^−3^, and 99.5 × 10^19^ cm^−3^ for the hybrid nanostructures obtained with 0.001 M, 0.005 M and 0.01 M of Zn(NO_3_)_2_, respectively. It can be appreciated that the values of N_D_ are considerably higher in the hybrid nanostructures synthesized with 0.005 M and 0.01 M of Zn(NO_3_)_2_ with respect to the TiO_2_ nanosponges. This fact may be related to a higher number of defects present in the nanostructures produced by the introduction of ZnO, thus improving the charge transfer of the nanostructures. Therefore, the nanostructures with higher photoelectrochemical activity, that is, the optimum nanostructures, will be for tungsten, the original and the hybrid with 0.005 M Mob, and for titanium, the hybrid nanostructure with 0.01 M Zn(NO_3_). This will be corroborated with the water splitting tests by photoelectrocatalysis.

#### 3.2.3. Water Splitting Tests

**Figure 7:**
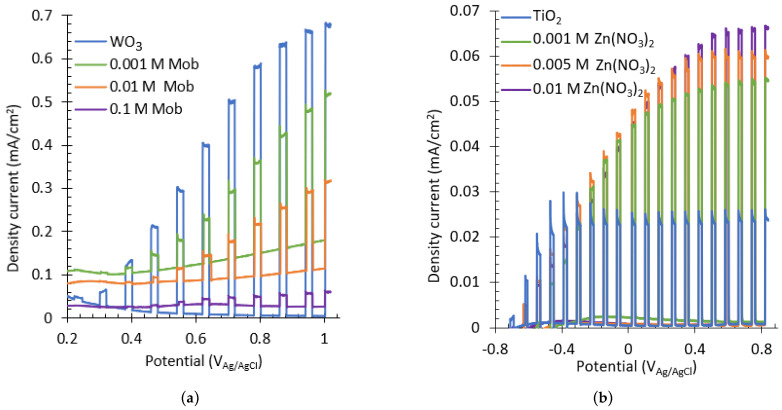
Water splitting curves of the (**a**) WO_3_ nanostructures synthesized with different Mob concentrations and (**b**) TiO_2_ nanostructures synthesized with different Zn(NO_3_)_2_ concentrations.

The influence of the doping element concentration in the electrolyte on the photoelectrochemical (PEC) behavior of the samples was also analyzed by water splitting tests, as shown in Figure 7. For WO_3_ nanostructures, it can be seen from Figure 7a that the sample with a higher photoresponse is the basic nanostructure with WO_3_ without adding Mob to the electrolyte. Furthermore, as the concentration of Mob increases, the photocurrent decreases, indicating worse photoelectrochemical properties. The worsening of the nanostructure after the addition of Mob could be explained by the fact that MoO_3_ is deposited on the surface of the nanostructure and prevents electron transfer.

On the other hand, from Figure 7b, it can be seen that the sample with the higher photocurrent is that with 0.01 M Zn(NO_3_)_2_, obtaining a photoelectrochemical response 141% higher than the crystalline TiO_2_ nanosponges. Furthermore, by increasing the Zn(NO_3_)_2_ concentration, the photoelectrochemical efficiency of the nanostructures is enhanced. This is due to the fact that ZnO crystals delay the recombination of the electron/hole pairs generated, increasing the lifetime of the excited electrons and thus enhancing the photocatalytic activity of the nanostructures.

### 3.3. Photoelectrocatalytic Degradation of Imazalil

#### 3.3.1. Imazalil Degradation in 0.1 M NaOH (pH = 13)

From all the characterization studies, it can be stated that the optimum nanostructure for W is the original one, without molybdenum. The nanostructure with W has already been proven to be an efficient photoanode for PEC degradation of emerging contaminants [19]. In contrast, for Ti, the original nanostructure could be enhanced, with the optimum nanostructure being the hybrid nanostructure TiO_2_-ZnO with 0.01 M ZnO. Consequently, in this study, the enhanced nanostructure of TiO_2_-ZnO is tested as a photoanode for PEC degradation of the pesticide Imazalil.

First, the hybrid TiO_2/_ZnO nanostructure with a Zn(NO_3_)_2_ concentration of 0.01 M is analyzed. Before carrying out the photoelectrocatalytic degradation of Imazalil, standards were examined by the UHPLC-MS-QTOF to obtain a calibration line that will permit us to calculate the concentration of the contaminant at any time. Figure S3 shows the EIC chromatogram of the standards in 0.1 M NaOH for the *m*/*z* value of 297.06, which corresponds to Imazalil.

Figure S3 shows how the intensity of the peak associated with Imazalil (*m*/*z* = 297.06) increases progressively as the concentration of Imazalil increases. With these results, it was possible to obtain a calibration line (Equation (2)) from which the exact concentration of Imazalil as a function of degradation time was calculated. Figure S4 (Supplementary Material) shows the calibration line of the area as a function of Imazalil concentration.

Area = 1,914,500 · Concentration (ppm) + 811,608(2)

**Figure 8:**
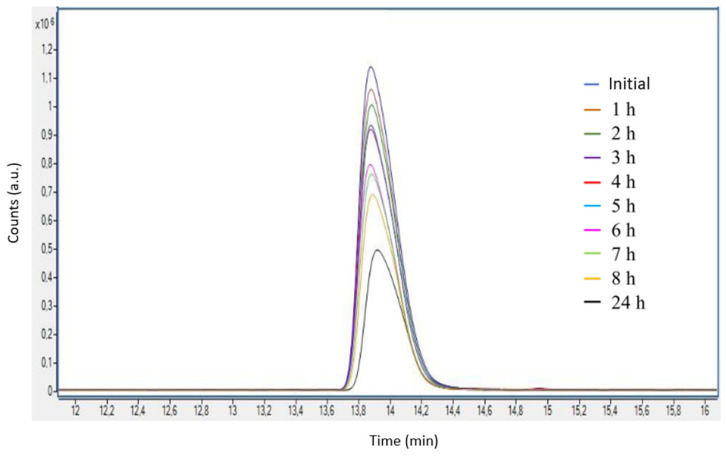
Enlarged EIC chromatograms of the peaks associated with Imazalil (*m*/*z* = 297.06) obtained during photoelectrocatalytic degradation of the compound in 0.1 M NaOH using hybrid TiO_2_-ZnO nanostructures as photoanodes.

Once the calibration line was determined, photoelectrocatalytic degradation of Imazalil in 0.1 M NaOH (pH = 13) was carried out under illumination conditions (AM 1.5) at a potential of 0.6 V _Ag/AgCl_ (3 M KCl) for 24 h using hybrid TiO_2_/ZnO nanostructures with a Zn(NO_3_)_2_ concentration of 0.01 M as photoanodes. Figure 8 shows the EIC chromatograms for an *m*/*z* = 297.06 obtained during the above photoelectrocatalytic degradation process.

Figure 8 shows that the concentration of Imazalil decreases over time. For a better analysis of the data, the percentages of Imazalil degradation as a function of time were calculated and are shown in Table 4. It can be seen that the concentration of Imazalil decreases over time until it reaches a concentration of 5 ppm after 24 h, which is equivalent to a degradation percentage of 49.8%.

**Figure 9:**
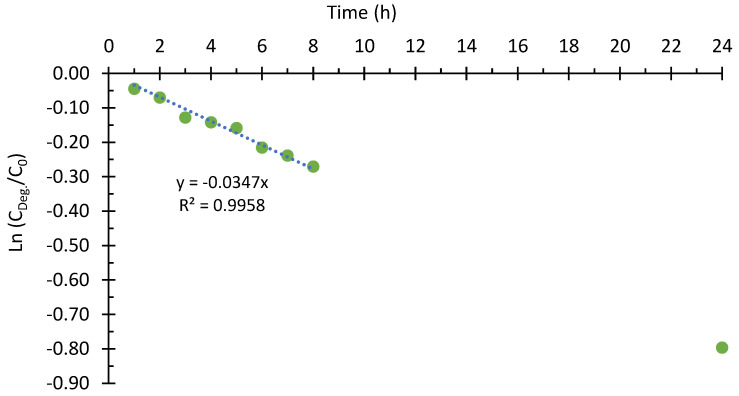
Fitting of pseudo-first-order kinetics for the PEC degradation of Imazalil in 0.1 M NaOH using hybrid TiO_2_-ZnO nanostructures as photoanodes.

Next, the kinetics of the photoelectrocatalytic degradation of Imazalil using hybrid TiO_2_-ZnO nanostructures as photoanodes are evaluated. In this case, as there are two reactants present in the degradation process (the contaminant and ·OH radicals), and one of them (·OH radicals) remains approximately constant during the experiment, it could be approximated to pseudo-first-order degradation kinetics. Therefore, representing the logarithm of the quotient between the concentration at each time and the initial concentration versus time (Figure 9) gives a straight line whose slope will provide the rate constant (k’) of the degradation reaction. Figure 9 shows a decreasing trend, and as the R2 obtained is greater than 0.99, it can be stated that the degradation kinetics of Imazalil follow pseudo-first-order kinetics (Equation (3)).

Furthermore, from Figure 9, it can be seen that the highest degradation rate takes place during the first three hours of degradation (12.1%). After that, the degradation rate decreases to values between 1 and 4% per hour. The high percentage of degradation obtained during the first hour could be due to the fact that the adsorption processes of Imazalil on the surface of the photocatalyst were carried out optimally. In this situation, the number of active sites present on the surface of the photocatalyst could be higher than the proportion of adsorbed molecules. However, as the degradation time advances, the nanostructure would become increasingly saturated due to the accumulation of adsorbed molecules on its surface, resulting in the system entering an equilibrium stage in which the degradation rate is given as a function of the adsorption and desorption processes of Imazalil on the surface of the photocatalysts. In addition, the kinetics may also be influenced by the presence of intermediate degradation compounds [53,54,55,56].

Ln (C/Co) = −0.0347·t(3)

According to Equation (3), the rate constant (k’) for the photoelectrocatalytic degradation reaction of Imazalil in 0.1 M NaOH using hybrid TiO_2_-ZnO nanostructures is 0.0347 h^−1^.

Although Imazalil was partially degraded in 0.1 M NaOH (pH = 13) using hybrid TiO_2_-ZnO nanostructures as photoanodes, the percentage of degradation obtained after 24 h (50%) was not as satisfactory as expected. The efficiency of PEC degradation is influenced by the pH of the solution, since it influences the adsorption and dissociation of the molecule to be degraded, the concentration of hydroxyl radicals, and the surface charges of the photocatalyst and the organic molecules. Consequently, it was decided to study the PEC degradation of Imazalil at different pHs in order to select the one that promotes the adsorption processes of Imazalil and thus increases the percentage of degradation.

#### 3.3.2. Effect of pH on Imazalil Degradation

The pKa value of pesticides marks their equilibrium state. When the pH increases above the pKa value of the pesticide, the pesticide is in its anionic state (A^−^), whereas if the pH is below the pKa value, the pesticide tends to be in its original state (AH), in the case of Imazalil, in the neutral state. Therefore, since the pKa value of Imazalil is 6.54 [57,58], it would be expected that the degradation of Imazalil would be favored at alkaline pHs. This is due to the fact that at pHs higher than the pKa of Imazalil (6.54), the pesticide is negatively charged and the photoelectrocatalyst is positively charged because of the application of the anodic potential. However, this occurs up to a certain limit where the pH is so high that there are too many negative charges (·OH) on the surface of the photocatalyst and the electrostatic repulsion between it and the negatively charged Imazalil increases. Therefore, as there is a counteracting effect, it was decided to perform a study on the photocatalytic degradation of Imazalil in a pH range between 5 and 13 (pHs 5, 6, 7, 8, 10, and 13) by adjusting the pH with NaOH and H_2_SO_4_. Lower pHs could not be used due to the instability of ZnO at acidic pHs [59]. Figure S3 (Supplementary Material) shows the degradation percentages obtained at different pHs for the PEC degradation of 10 ppm of Imazalil during 6 h at 0.6 V_Ag/AgCl_ (3 M KCl) using hybrid TiO_2_-ZnO nanostructures as photoanodes. The results obtained during the study of the influence of pH on the photoelectrocatalytic degradation of Imazalil (Figure S5) confirmed that the efficiency of the photocatalyst is linked to the pH of the medium. Figure S5 shows that increasing the pH of the solution from 5 to 6 considerably increases the percentage of degradation of Imazalil (from 21% to 41%), while remaining practically constant in the pH range between 6 and 8. However, by increasing the pH of the medium above 8, the percentage of degradation begins to decrease until reaching a value of 28% for pH = 13. After completing this series of tests, it was concluded that the photoelectrocatalytic degradation of pesticides is favored when the organic molecule to be degraded is negatively charged, i.e., at pHs above the pKa of the pesticide, since the photoelectrocatalyst will be positively charged by the effect of applying an anodic potential. According to these results, it was decided to carry out the photoelectrocatalytic degradation of Imazalil at a pH between 6 and 8, at which the hybrid TiO_2_-ZnO nanostructures work optimally.

On the other hand, instead of using NaOH as an electrolyte, it has been demonstrated in different publications that the addition of sulfate ions improves the degradation process of pesticides [60,61]. This is because the redox potential of sulfate radicals (2.6–3.1 eV) is higher than that of hydroxyl radicals (1.8–2.7 eV), which makes the degradation of organic compounds more effective in the presence of sulfate radicals.

#### 3.3.3. Imazalil Degradation in 0.1 M Na_2_SO_4_ (pH = 6.2)

Consequently, once the optimum medium was determined, another PEC degradation of Imazalil was carried out in 0.1 M sodium sulfate (Na_2_SO_4_) in order to test the efficiency of the process at pH 6.2. First, standards of Imazalil in 0.1 M Na_2_SO_4_ were analyzed by the UHPLC-MS-QTOF in order to obtain the calibration line (Figure S6, see Supplementary Material) and the equation to be able to evaluate the concentration of Imazalil in 0.1 M Na_2_SO_4_ as a function of the intensity recorded (Equation (4)).

Area = 2,538,010·Concentration (ppm) + 1,161,910(4)

**Figure 10:**
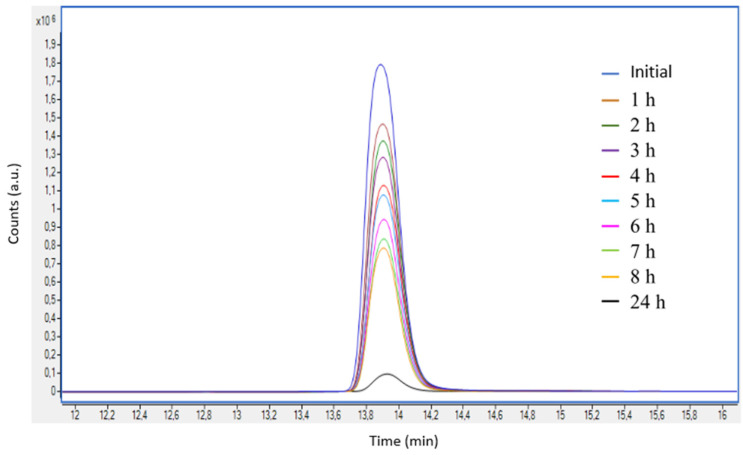
EIC chromatograms of the peaks associated with Imazalil (*m*/*z* = 297.06) obtained during photoelectrocatalytic de-gradation of the compound in 0.1 M Na_2_SO_4_ using hybrid TiO_2_-ZnO nanostructures as photoanodes.

Figure 10 shows the EIC chromatograms for an m/z = 297.06 obtained during the PEC degradation process in 0.1 M Na_2_SO_4_. Figure 10 shows how the area of the EIC chromatogram of Imazalil decreases over time until it is practically not visible after 24 h. This indicated that under these photoelectrocatalytic degradation conditions, the concentration of Imazalil decreases markedly over time and is almost eliminated after 24 h of PEC.

Table 5 shows the degradation percentages obtained from Equation (3) during the photoelectrocatalytic degradation of 10 ppm Imazalil in 0.1 M Na_2_SO_4_ using hybrid TiO_2_-ZnO nanostructures as photoanodes. From Table 5, it can be seen that the concentration of Imazalil decreases considerably over time during the 24 h of the test until it reaches a degradation percentage of 99.1%. During the first hour of the test, the degradation percentage increases rapidly (13.3%). However, once the system is stabilized, the degradation percentage increases more slowly. Again, this phenomenon could be due to the fact that after one hour, the photocatalyst surface is saturated and most of the active sites present in the nanostructures are occupied. Therefore, after one hour of testing, the degradation was controlled by the adsorption and desorption processes of Imazalil on the surface of the photocatalyst.

**Figure 11:**
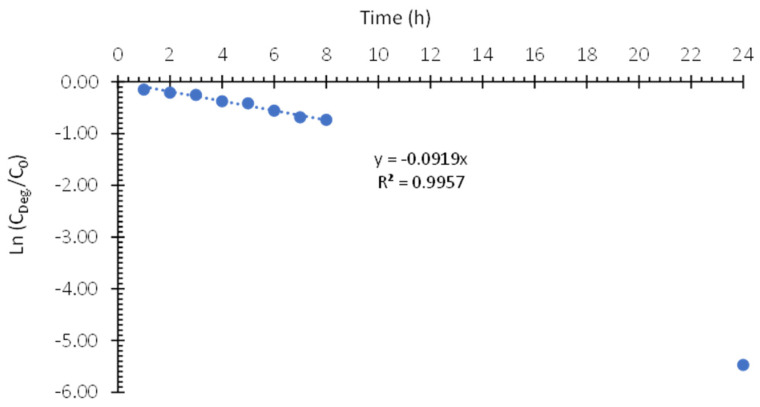
Fitting of pseudo-first-order kinetics for the PEC degradation of Imazalil in 0.1 M Na_2_SO_4_ using hybrid TiO_2_-ZnO nanostructures as photoanodes.

Next, the kinetics constant of the PEC degradation was determined by plotting the ln(C/C_o_) versus time, following a pseudo-first-order kinetics. Figure 11 shows the line from which the rate constant (k’) could be obtained. In this case, in order to achieve a better fit, the concentration value obtained at 24 h has been omitted since, being practically zero, it did not fit the kinetic equation well. Equation (5) shows that the rate constant for the PEC degradation of Imazalil in Na_2_SO_4_ 0.1 M using TiO_2_-ZnO hybrid nanostructures as photoanodes is 0.0919 h^−1^ (0.0015 min^−1^) “Comparing our degradation parameters with those obtained with other advanced oxidation processes to remove found in literature, it can be affirmed that the TiO_2_-ZnO hybrid nanostructures obtained in this study are very efficient for Imazalil removal [62,63,64]”.

Ln (C/Co) = −0.0919·t (5)

As expected, performing the PEC degradation of Imazalil in 0.1 M Na_2_SO_4_ instead of 0.1 M NaOH using hybrid TiO_2_-ZnO nanostructures as photoanodes leads to a significant increase in the percentage of degradation; namely, the percentage of degradation increased from 49.8% in 0.1 M NaOH to 99.1% in 0.1 M Na_2_SO_4_ after 24 h. Furthermore, the degradation rate in 0.1 M Na_2_SO_4_ (k’ = 0.0919 h^−1^) was approximately 186% higher than that obtained in NaOH 0.1 M (k’ = 0.0347 h^−1^). The better performance of the hybrid nanostructures in 0.1 M Na_2_SO_4_ can be attributed to two factors. The first factor is an increase in the adsorption of Imazalil on the surface of the photocatalyst. As it was previously verified, by carrying out the photoelectrocatalytic degradation of Imazalil at optimum pH (pH = 6.2), the adsorption processes of Imazalil on the surface of the hybrid nanostructures are considerably improved [53,54,55,56]. The second factor is the presence of sulfate ions in solution. As previously commented, the presence of sulfate ions allows the formation of sulfate radicals whose redox potential (2.6–3.1 V) is higher than that of hydroxyl radicals (1.8–2.7 V), thus increasing the degradation rate of Imazalil [55,61].

#### 3.3.4. Intermediate Degradation Products and Degradation Mechanism

Finally, a study on the reaction intermediates and possible routes of PEC degradation of Imazalil was performed under the selected optimal conditions: PEC degradation of Imazalil in Na_2_SO_4_ 0.1 M using the hybrid TiO_2_-ZnO nanostructure. The reaction intermediates were identified by UHPLC-MS-QTOF from the degraded samples and from literature [53,55,58,61,65,66,67]. Table 6 shows the reaction intermediates that could be identified by UHPLC-MS-QTOF, their *m*/*z* value, and their retention time.

Intermediate 1 is given by the fragmentation of Imazalil without the presence of the imidazole ring (*m*/*z* = 69), but with a nitrogen atom in its place. As intermediate 1, intermediate 2 was identified as the fragmentation of Imazalil with a loss of *m*/*z* = 41, corresponding to the compound CH_2_-CH=CH_2_. Next, intermediate 3 is similar to intermediate 2, but with the addition of a hydroxyl radical. Intermediates 4 and 5 are monohydroxylated compounds derived from Imazalil. Finally, intermediates 6 and 7 are dihydroxylated products; intermediate 6 possesses both hydroxyls on the benzene ring, while intermediate 7 possesses them on the double bond of the compound. Figure S7 (Supplementary Material) shows the EIC chromatograms for each of the intermediates identified.

**Figure 12:**
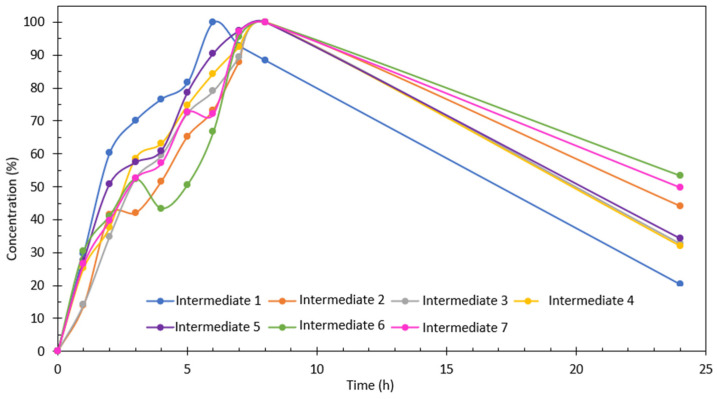
Evolution of the intermediate’s concentration during the degradation of Imazalil in 0.1 M Na_2_SO_4_ using the hybrid TiO_2_-ZnO nanostructure as photoanode.

Figure S7a shows that the concentration of intermediate 1 increases as the degradation time progresses until it reaches a maximum concentration at 6 h, after which the concentration of intermediate 1 begins to decrease until it practically disappears after 24 h. Figure S7b shows that the concentration of intermediate 2 increases as the degradation process progresses until it reaches a maximum value after 8 h and decreases at 24 h. Figure S7c–g shows the concentrations of intermediates 3, 4, 5, 6, and 7, respectively. The tendency of all of them is similar: the concentration increases as the degradation time increases, reaching a maximum at 8 h and finally decreasing at 24 h. Furthermore, the tendency of the concentration of each intermediate has been estimated from the areas of the peaks obtained from the EIC chromatogram (Figure 12). The concentration is quantified as a percentage, with the sample with the largest area being 100%. Figure 12 shows that the intermediate compounds that formed the fastest were intermediates 1 and 5, while the slowest were intermediates 2, 6, and 7. On the other hand, the intermediate compound whose degradation was fastest and had the highest degradation rate was also intermediate 1, with intermediates 6 and 7 being the slowest to degrade and those that obtained the lowest degradation rate after 24 h.

**Figure 13:**
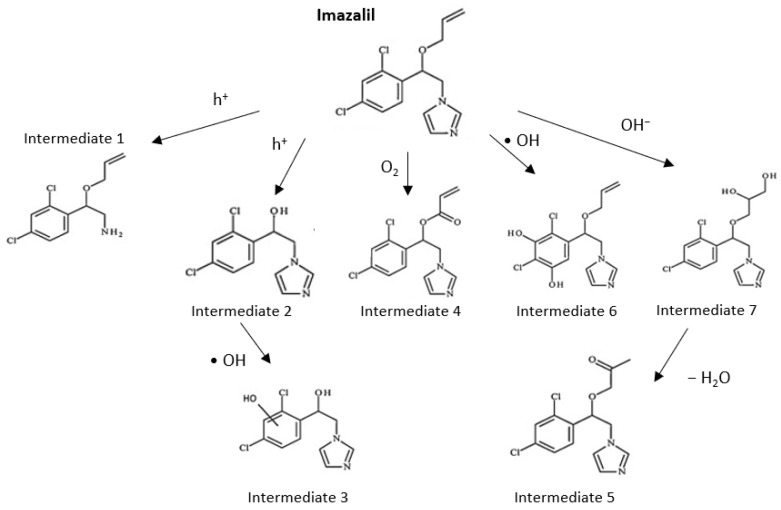
Degradation route of Imazalil with intermediate compound found in the PEC degradation with hybrid TiO_2_-ZnO nanostructure in Na_2_SO_4_ 0.1 M.

Finally, based on the intermediates identified during the PEC degradation of Imazalil in 0.1 M Na_2_SO_4_ for 24 h using TiO_2_-ZnO hybrid nanostructures as photoanodes, a possible degradation route was proposed (Figure 13).

The mechanism proposed in Figure 13 can be divided into two processes: processes by which Imazalil is attacked by holes (h^+^), which give rise to smaller molecules; and processes by which Imazalil is attacked by hydroxyl radicals (·OH), obtaining Imazalil derivatives with a higher molecular weight than the starting compound. Intermediate 1 is produced by the attack of Imazalil by photogenerated holes that produce the separation of the imidazole ring from the rest of the molecule. This degradation mechanism is the most favorable since intermediate 1 is the compound that is formed the fastest. Instead, if the attack occurs on the double bond of Imazalil, the compound will fragment, losing an *m*/*z* of 41 corresponding to CH_2_−CH=CH_2_, giving rise to intermediate 2, which can suffer the attack of hydroxyl radicals on the benzene ring to form intermediate 3.

On the other hand, when the initial attack is produced by hydroxyl radicals, intermediates 4, 5, 6, and 7 will be formed. Intermediate 4 is formed by the transformation of the ether into a ketone by the attack of a hydroxyl radical. Intermediate 6 is formed from the dihydroxylation of the benzene ring. Intermediate 7 is formed by dihydroxylation of the double bond. Intermediate 5 is formed from intermediate 7 by addition of oxygen, followed by a loss of H_2_O^•^ to finally carry out a transformation from alcohol to ketone [58,61].

## 4. Conclusions

In this research, different types of nanostructures (WO_3_ and TiO_2_) have been synthesized by anodization of W and Ti, respectively, under hydrodynamic conditions. Next, hybrid nanostructures have also been synthesized with WO_3_-MoO_3_ and TiO_2_-ZnO to improve the original nanostructures. In the case of WO_3_-MoO_3_, the photocatalytic activity of the nanostructures could not be increased by the electrodeposition of MoO_3_ on the surface of the WO_3_ nanostructures. In contrast, the photocatalytic activity of TiO_2_-ZnO was significantly enhanced compared to TiO_2_ nanosponges. The optimum nanostructure was achieved when performing the ZnO electrodeposition with a 0.01 M Zn(NO_3_)_2_ concentration, obtaining a photoelectrochemical response 141% higher compared to the crystalline TiO_2_ nanosponges. These enhanced nanostructures were used as a photoanode for PEC degradation of the pesticide Imazalil. It has been proven that the percentage of degradation varies depending on the pH of the medium, obtaining an optimal pH for PEC degradation of Imazalil at pHs between 6 and 8. A degradation percentage of 99.1% was obtained by carrying out the PEC degradation of 10 ppm of Imazalil in 0.1 M Na_2_SO_4_ (pH 6.2) for 24 h at a potential of 0.6 V_Ag/AgCl_ (3 M KCl). Furthermore, intermediates of degradation and the degradation route have been found.

## Figures and Tables

**Table 1:** Results of the EDX analysis shown as percentage, in weight and atomic, for the different elements present in the samples.

Concentration of Mob (M)	% (Weight)			% (Atomic)			Concentration of Zn(NO_3_)_2_ (M)	% Weight			% Atomic		
	O	W	Mo	O	W	Mo		O	Ti	Zn	O	Ti	Zn
0	18.02	81.98	0.00	71.66	28.33	0.00	0	34.51	65.49	0.00	61.21	38.79	0.00
0.001	28.77	70.32	0.92	82.10	17.46	0.44	0.001	38.11	57.56	4.33	65.26	32.92	1.82
0.01	19.40	78.88	1.71	73.07	25.85	1.08	0.005	36.07	58.08	5.85	63.39	34.10	2.51
0.1	18.01	80.02	1.97	71.18	27.53	1.30	0.01	35.04	57.81	7.15	62.45	34.43	3.12

**Table 2:** Resistance values of the different samples.

Concentration of Mob (M)	R_1_ (Ω·cm^2^)	R_2_ (Ω·cm^2^)	Concentration of Zn(NO_3_)_2_ (M)	R_1_ (Ω·cm^2^)	R_2_ (Ω·cm^2^)
0.000	14	2355	0.000	4492	20,491
0.001	26	3830	0.001	5823	14,184
0.010	15	2095	0.005	221	5520
0.10	26	7283	0.010	28	3710

**Table 3:** Donor density values obtained from Mott-Schottky test for the different samples.

Concentration of Mob (M)	N_D_·10^19^ (cm^−3^)	Concentration of Zn(NO_3_)_2_ (M)	N_D_·10^19^ (cm^−3^)
0.000	297.4	0.000	1.2
0.001	54.0	0.001	2.1
0.010	144.1	0.005	85.8
0.100	37.1	0.010	99.5

**Table 4:** Results of photoelectrocatalytic degradation of Imazalil in 0.1 M NaOH over time using TiO_2_-ZnO hybrid nanostructures as photoanodes.

Time (h)	Concentration (ppm)	Degraded Concentration (ppm)	Degradation (%)
0	10.00	0.00	0.0
1	9.56	0.44	4.4
2	9.32	0.68	6.8
3	8.79	1.21	12.1
4	8.67	1.33	13.3
5	8.53	1.47	14.7
6	8.06	1.94	19.4
7	7.87	2.13	21.3
8	7.63	2.37	23.7
24	5.02	4.98	49.8

**Table 5:** Results of photoelectrocatalytic degradations of Imazalil in 0.1 M Na_2_SO_4_ over time using TiO2-ZnO hybrid nanostructures as photoanodes.

Time (h)	Concentration (ppm)	Degraded Concentration (ppm)	Degradation (%)
0	10.0	0.00	0.0
1	8.67	1.33	13.3
2	8.16	1.84	18.4
3	7.81	2.19	21.9
4	6.90	3.10	31.0
5	6.62	3.38	33.8
6	5.75	4.25	42.5
7	5.07	4.93	49.3
8	4.82	5.18	51.8
24	0.09	9.91	99.1

**Table 6:** Degradation intermediates obtained during the PEC degradation of Imazalil in 0.1 M Na_2_SO_4_ using the hybrid TiO_2_-ZnO nanostructure as photoanode.

Intermediate Number	Name	Chemical Structure	*m*/*z*	Retention Time (min)
1	2-(aliloxi)-2-(2,4-diclorofenil)etanamina	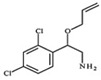	246.05	12.6
2	1-(2,4-diclorofenil)-2-(1H-imidazol-1-il)etanol	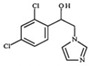	257.02	9.6
3	2,6-dicloro-3-(1-hidroxi-2-(1H-imidazol-1-il)etil)fenol	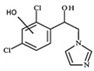	273.06	13.2
4	acrilato de 1-(2,4-diclorofenil)-2-(1H-imidazol-1-il)etilo	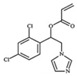	311.04	12.6
5	1-(1-(2,4-diclorofenil)-2-(1H-imidazol-1-il)etoxi)propan-2-ona	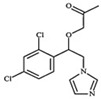	313.03	12.6
6	5-(1-(aliloxi)-2-(1H-imidazol-1-il)etil)-2,4-diclorobenceno-1,3-diol	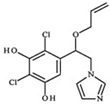	329.04	9.8
7	3-(1-(2,4-diclorofenil)-2-(1H-imidazol-1-il)etoxi)propano-1,2-diol	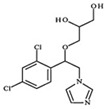	331.06	9.8

## Data Availability

Data available on request.

## Supplementary Materials

The following supporting information can be downloaded at: https://www.mdpi.com/article/10.3390/nano13182584/s1. Figure S1: Scheme of PEC mechanism; Table S1: Average crystal size, expressed as D (nm), for the different studied samples: WO_3_, WO_3_-Mo, TiO_2_ and TiO_2_-ZnO; Figure S2: Equivalent circuit for the nanostructures synthesized; Figure S3: EIC chromatogram for the Imazalil standards in 0.1 M NaOH and inset of the magnification of the peak. Figure S4: Calibration line for Imazalil standards in 0.1 M NaOH solution obtained with UHPLC-MS-QTOF; Figure S5: Influence of pH on the percentage of PEC degradation of Imazalil; Figure S6: Calibration line for Imazalil standards in 0.1 M Na_2_SO_4_ solution obtained with UHPLC-MS-QTOF; Figure S7: EIC chromatograms for (a) Intermediate 1, *m*/*z* = 246.05 (b) Intermediate 2 *m*/*z* = 257.02 (c) Intermediate 3, *m*/*z* = 273.06 (d) Intermediate 4, *m*/*z* = 311.04 (e) Intermediate 5, *m*/*z* = 313.03 (f) Intermediate 6, *m*/*z* = 329.04, and (g) Intermediate 7, *m*/*z* = 331.06.
