# Supplementary material for: Characterization and Comparison of WO3/WO3-MoO3 and TiO2/TiO2-ZnO Nanostructures for Photoelectrocatalytic Degradation of the Pesticide Imazalil
Source: Nanomaterials (Basel). 2023 Sep 18;13(18):2584. doi: 10.3390/nano13182584 (PMC10535956; doi:10.3390/nano13182584)
Supplement: Supplementary file 1 [file nanomaterials-13-02584-s001.zip › nanomaterials-2597613-supplementary.pdf]

Supplementary Materials

Characterization and Comparison of WO_3/WO_3-MoO_3 and TiO_2/TiO_2-ZnO Nanostructures for Photoelectrocatalytic Degradation of the Pesticide Imazalil

Mireia Cifre-Herrando, Gemma Roselló-Márquez, Pedro José Navarro-Gázquez, María José Muñoz-Portero, Encarnación Blasco-Tamarit and José García-Antón *

Ingeniería Electroquímica y Corrosión (IEC), Instituto Universitario de Seguridad Industrial, Radiofísica y Medioambiental (ISIRYM), Universitat Politècnica de València, C/Camino de Vera s/n, 46022 Valencia, Spain
* Correspondence: jgarciaa@iqn.upv.es

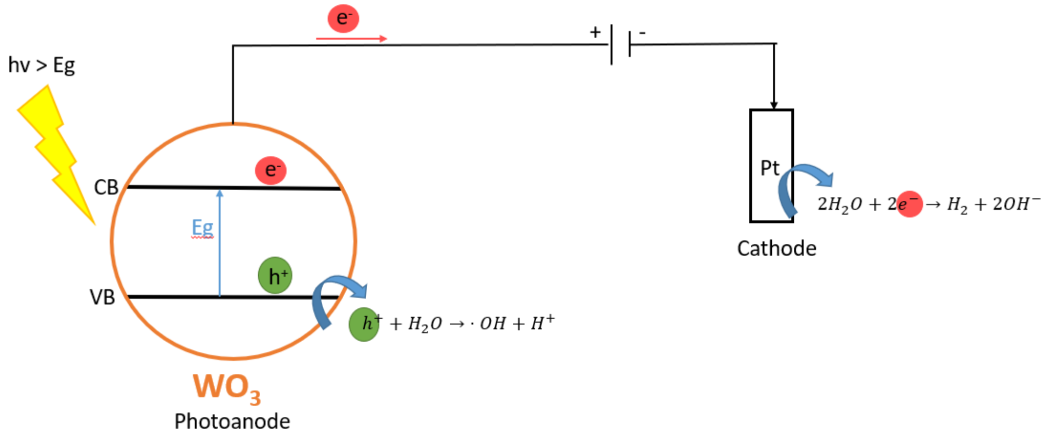

Figure S1. Scheme of PEC mechanism.

Table S1. Average crystal size, expressed as D (nm), for the different studied samples: WO_3, WO_3-Mo, TiO_2 and TiO_2-ZnO.

Sample	Peak		D (nm)
	Associated to	Plane	
WO_3-	WO_3-	020	43.3
WO_3-Mo	MoO_3	110	43.2
	WO_3	020	40.4
TiO_2	TiO_2	101	26.2
TiO_2-ZnO	TiO_2	101	25.5
	ZnO	101	44.2

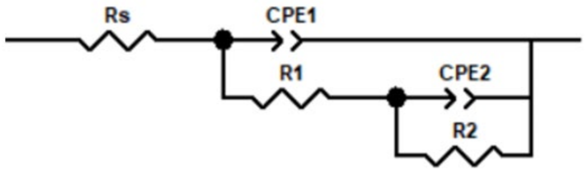

Figure S2. Equivalent circuit for the nanostructures synthesized.

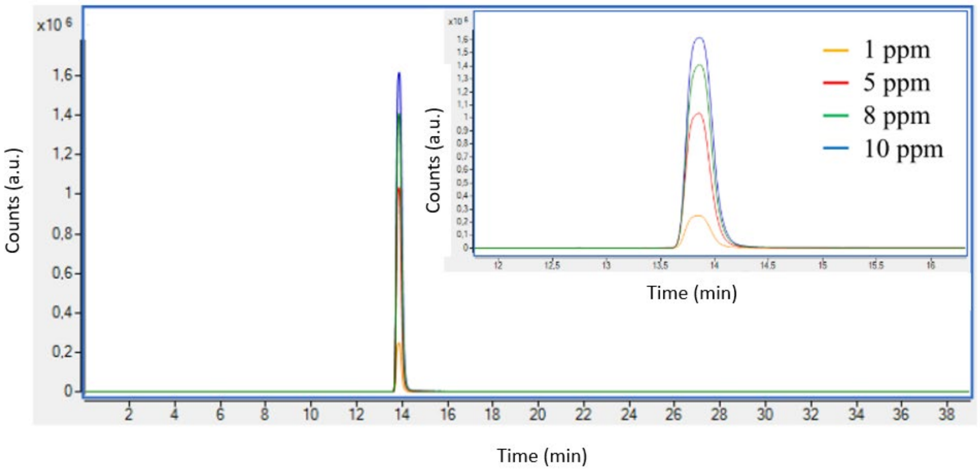

Figure S3. EIC chromatogram for the Imazalil standards in 0.1 M NaOH and inset of the magnification of the peak.

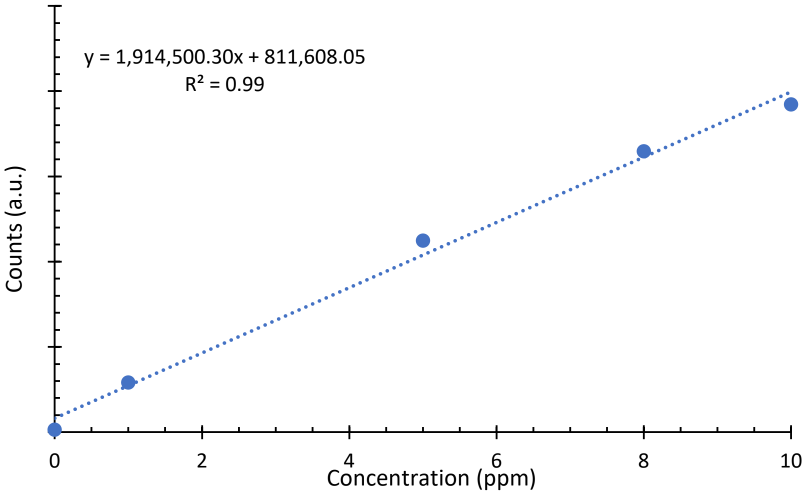

Figure S4. Calibration line for Imazalil standards in 0.1 M NaOH solution obtained with UHPLC-MS-QTOF.

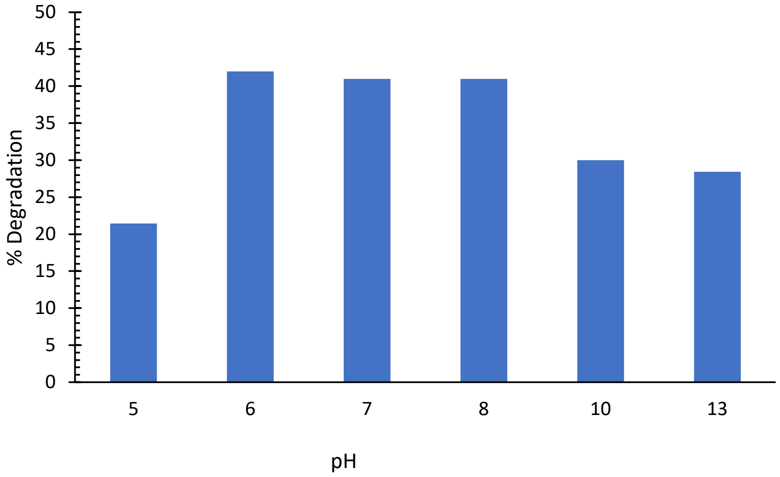

Figure S5. Influence of pH on the percentage of PEC degradation of Imazalil.

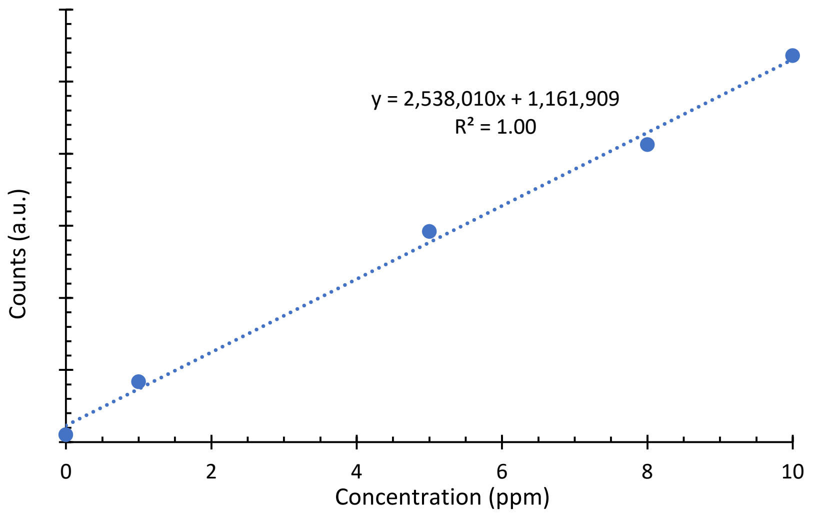

Figure S6. Calibration line for Imazalil standards in 0.1 M Na_2SO_4 solution obtained with UHPLC-MS-QTOF.

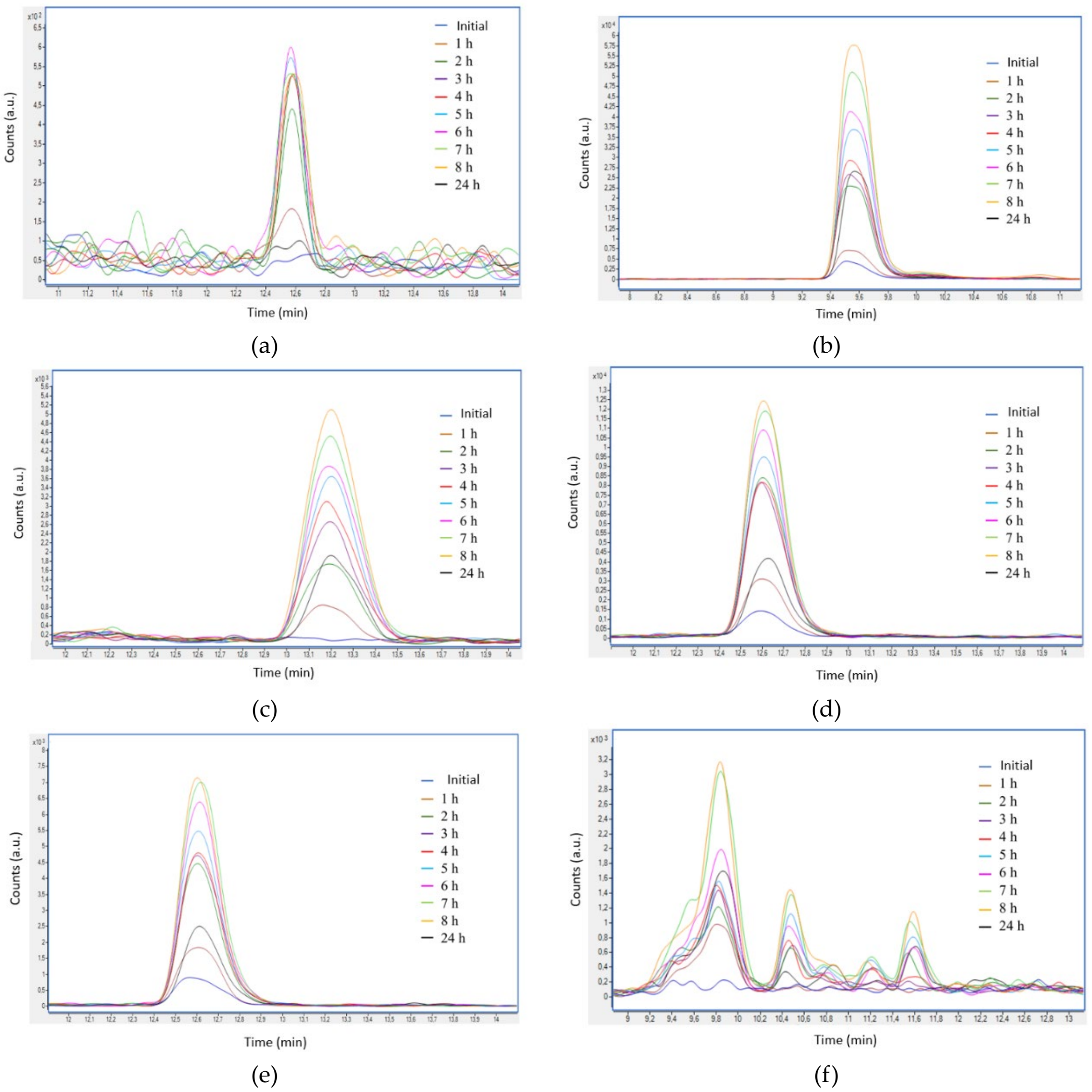

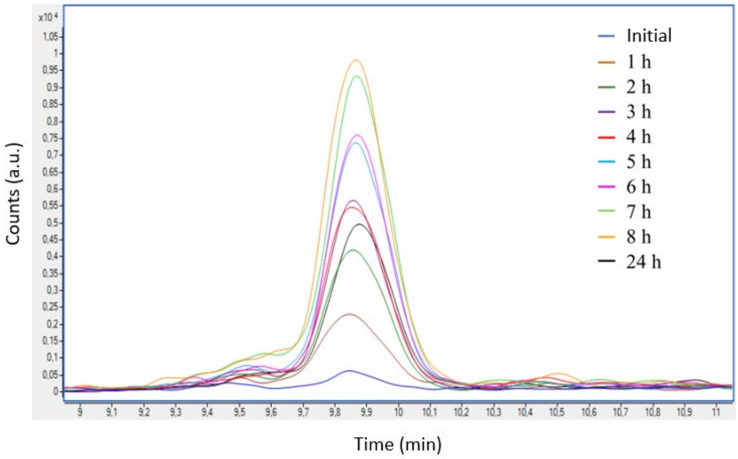

(g)

Figure S7. EIC chromatograms for (a) Intermediate 1, m/z = 246.05 (b) Intermediate 2 m/z = 257.02 (c) Intermediate 3, m/z = 273.06 (d) Intermediate 4, m/z = 311.04 (e) Intermediate 5, m/z = 313.03 (f) Intermediate 6, m/z = 329.04 and (g) Intermediate 7, m/z = 331.06 obtained during the PEC degradation of Imazalil in 0.1 M Na2SO4 using the hybrid TiO_2-ZnO nanostructure as photoanode.
